# First Outbreak Response Using an Oral Cholera Vaccine in Africa: Vaccine Coverage, Acceptability and Surveillance of Adverse Events, Guinea, 2012

**DOI:** 10.1371/journal.pntd.0002465

**Published:** 2013-10-17

**Authors:** Francisco J. Luquero, Lise Grout, Iza Ciglenecki, Keita Sakoba, Bala Traore, Melat Heile, Alpha Amadou Dialo, Christian Itama, Micaela Serafini, Dominique Legros, Rebecca F. Grais

**Affiliations:** 1 Epicentre, Paris, France; 2 Médecins sans Frontières, Geneva, Switzerland; 3 Ministry of Health, Conakry, Guinea; 4 African Cholera Surveillance Network, Paris, France; 5 Direction Préfectorale de la Santé, Ministry of Health, Conakry, Guinea; 6 Médecins sans Frontières, Conakry, Guinea; 7 Section de Recherche, Ministry of Health, Conakry, Guinea; 8 World Health Organization, Conakry, Guinea; Massachusetts General Hospital, United States of America

## Abstract

**Background:**

Despite World Health Organization (WHO) prequalification of two safe and effective oral cholera vaccines (OCV), concerns about the acceptability, potential diversion of resources, cost and feasibility of implementing timely campaigns has discouraged their use. In 2012, the Ministry of Health of Guinea, with the support of Médecins Sans Frontières organized the first mass vaccination campaign using a two-dose OCV (Shanchol) as an additional control measure to respond to the on-going nationwide epidemic. Overall, 316,250 vaccines were delivered. Here, we present the results of vaccination coverage, acceptability and surveillance of adverse events.

**Methodology/Principal Findings:**

We performed a cross-sectional cluster survey and implemented adverse event surveillance. The study population included individuals older than 12 months, eligible for vaccination, and residing in the areas targeted for vaccination (Forécariah and Boffa, Guinea). Data sources were household interviews with verification by vaccination card and notifications of adverse events from surveillance at vaccination posts and health centres. In total 5,248 people were included in the survey, 3,993 in Boffa and 1,255 in Forécariah. Overall, 89.4% [95%CI:86.4–91.8%] and 87.7% [95%CI:84.2–90.6%] were vaccinated during the first round and 79.8% [95%CI:75.6–83.4%] and 82.9% [95%CI:76.6–87.7%] during the second round in Boffa and Forécariah respectively. The two dose vaccine coverage (including card and oral reporting) was 75.8% [95%CI: 71.2–75.9%] in Boffa and 75.9% [95%CI: 69.8–80.9%] in Forécariah respectively. Vaccination coverage was higher in children. The main reason for non-vaccination was absence. No severe adverse events were notified.

**Conclusions/Significance:**

The well-accepted mass vaccination campaign reached high coverage in a remote area with a mobile population. Although OCV should not be foreseen as the long-term solution for global cholera control, they should be integrated as an additional tool into the response.

## Author Summary

Two safe and effective oral cholera vaccines are recommended by the World Health Organization for cholera prevention and control; however, concerns about the acceptability, potential diversion of resources, cost and feasibility of implementing timely campaigns has discouraged their use. In 2012, the Ministry of Health of Guinea, with the support of Médecins Sans Frontières, organized the first mass vaccination campaign using a two-dose oral cholera vaccine (Shanchol) as an additional control measure to respond to an on-going nationwide epidemic. This was also the first time that Shanchol was used in a mass vaccination campaign on the African continent. High coverage was reached within a few weeks, and the campaigns were well accepted by the population. Synergies between different axes in cholera control interventions should be pursued as described here, and although oral cholera vaccines should not be foreseen as the long-term solution for global cholera control, they should be integrated as an additional tool into the outbreak response strategies.

## Introduction

Provision of safe water and proper sanitation are without doubt the long-term and only solution for cholera control [1], [2]. However, controlling cholera globally is far from being achieved; the disease burden is increasing with large-scale outbreaks reported in the past several years, such as those in Haiti and Zimbabwe [3]. Current outbreak response interventions focus on case management and access to health care, as well as the immediate provision of safe water and hygiene promotion [1]. However, current outbreak control activities have proven insufficient to avoid massive numbers of cases and deaths in recent large-scale outbreaks. The adequate treatment of cases for example, although crucial to decrease mortality, has a limited impact in controlling disease spread [1], [3]. Oral cholera vaccines (OCV), which have the potential to reduce the number of cases and minimize the spread of disease [4], [5], could be an important addition to the cholera response arsenal [1], [6], [7].

The World Health Organization (WHO) prequalifies the OCV Dukoral (SBL Vaccine/Crucell, Sweden) and Shanchol (ShantaBiotechnics, Hyderabad, India). Both are killed whole cell *V. cholerae* O1 vaccines; Shanchol also contains *V. cholerae* O139 and Dukoral the recombinant cholera toxin B subunit. The two vaccines share a good safety and efficacy profile with an estimated protection of 60–85% for 2–3 years [1]. Although, recommended by WHO (including in response to outbreaks since 2010) [8], their use as public health tools has been limited. Specifically, questions about the acceptability, feasibility, cost and potential diversion of resources have discouraged the use of OCV for outbreak control [9].

In 2012, the Ministry of Health (MoH) of Guinea, with the support of Médecins Sans Frontières-Operational Centre Geneva (MSF) organized the first cholera outbreak response in Africa using an OCV in the Republic of Guinea (Guinea). This was also the first time that Shanchol was used in a mass vaccination campaign on the African continent. Cholera has been reported in Guinea since 1970. The largest outbreak was in 1994 with more than 30,000 cases and 670 deaths reported. The most affected areas were the coastal prefectures and the islands (Maritime Guinea, where the capital Conakry is located) [10]. From 2003 to 2007, cholera outbreaks were reported each year during the rainy season (July–August) throughout the country with Maritime Guinea remaining the most affected area. From 2008 to 2011, only sporadic cases were reported [11].

In 2012, the first cholera cases were reported in Forécariah (Maritime Guinea) before the rainy season. From February 2 to March 8, a total of 147 cases and 13 deaths were reported. On March 3, the first case was reported and confirmed in Conakry. A cholera outbreak was also on going in neighbouring Sierra Leone, with 13,934 cases and 232 deaths reported countrywide between January and August 2012 [12]. The regional nature of the epidemic, the early notification of cases before the peak of the rainy season and the long interval without outbreaks, thereby increasing the number of susceptible individuals due to lack of prior exposure, all suggested the possibility of a large epidemic in Guinea in 2012.

**Figure 1:**
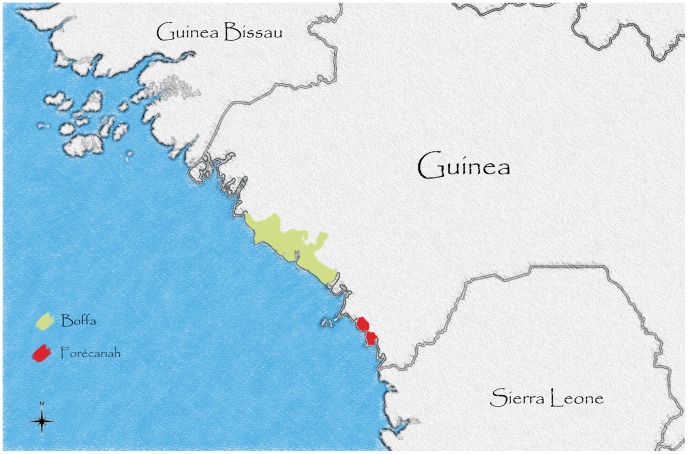
Target areas by the non-selective mass vaccination campaigns, Guinea, 2012.

**Figure 2:**
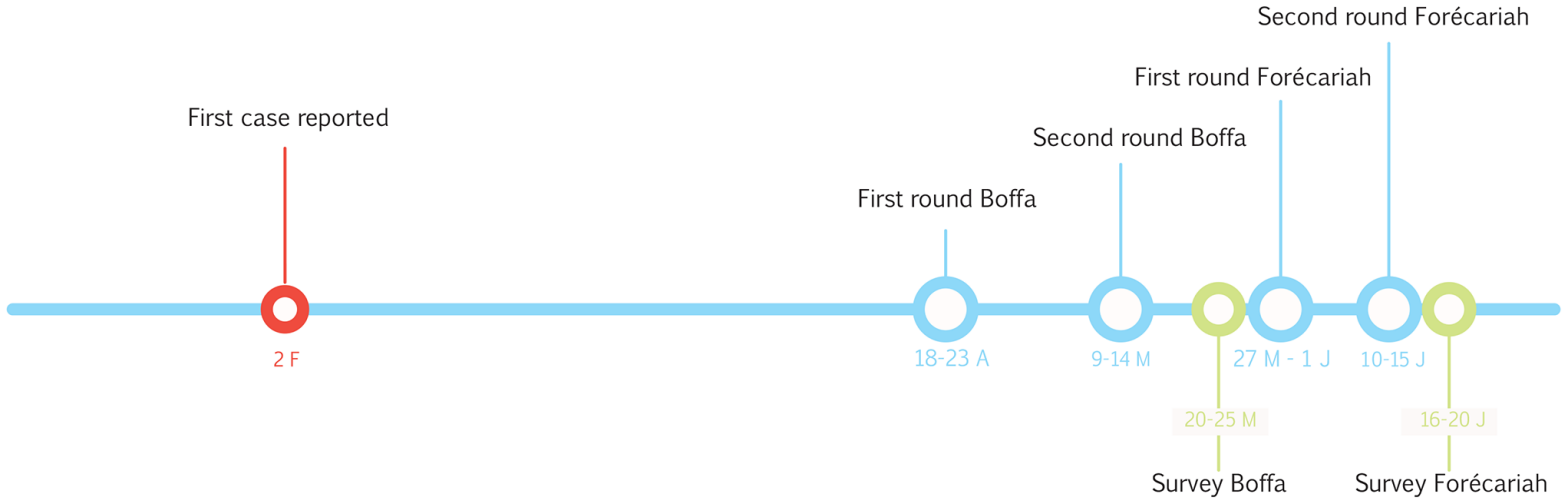
Timeline of the cholera vaccination campaigns and implementation of the field surveys in Guinea in 2012. Months are abbreviated as follows: F = February, A = April, M = May, J = June.

Case management, water, health education, hygiene and sanitation interventions were implemented in response to the outbreak. Non-selective mass vaccination campaigns were implemented in the prefectures of Boffa and Forécariah (Figure 1). Two doses of Shanchol, two weeks apart were offered from April 18 to May 14, 2012 in Boffa and from May 27 to June 15, 2012 in Forécariah (Figure 2). Overall, 316,250 vaccines were delivered by 43 teams (of 9 members in Boffa and 5 in Forécariah) in 287 vaccination sites (one per village or settlement). All individuals older than 12 months were eligible for vaccination in both rounds. Pregnant women were offered vaccine after a careful examination of the risk and benefits (an on-going outbreak in a remote rural place with limited access to health care and high cholera associated mortality in the past) following the manufacture and WHO recommendations [8]. Vaccines were stored under cold chain, but were transported and used at ambient temperature on vaccination days. Before administration, vaccine vial temperature monitor was checked for stability and all remained valid.

Here, we present the results of household-based vaccination coverage and acceptability surveys and surveillance of adverse events.

## Methods

### Cross-Sectional Survey

All individuals older than 12 months, resident in the six sub-prefectures bordering the sea in Boffa prefecture (Koba, Boffa-centre, Douprou, Tougnifily, and part of Mankountan and Tamita) and in the sub-prefectures of Kaback and Kakossa in Forécariah prefecture were targeted for vaccination and were eligible for inclusion in the survey (Figure 1).The coastal area of Boffa combines both inland areas and several islands. Kaback and Kakossa are two separate islands. Residents were defined as persons living (sleeping and eating) in the area for at least the previous two weeks. The adult population is mobile with men in particular, leaving and returning to the area for fishing, agriculture and trade.

A representative sample of the population in each survey site (Boffa and Forécariah) was selected using cluster-based sampling with population proportional to size [13]. To sample households within the selected sectors, all households were enumerated. The first household was selected with the aid of a random number table and subsequent households were selected by proximity (first household to the left). In the urban area of Boffa and in Kaback Island in Forécariah, satellite-map based sampling was used to select randomly the starting point of the cluster [14]. This methodology was used in urban Boffa because of the large number of households to enumerate and in Kaback Island because of the absence of accurate population data per sector.

The sample size was calculated to obtain a representative estimate of the proportion of residents who received two doses of OCV by age group (1–4, 5–14, 15 years and older). Sample size was calculated to ensure a sufficiently precise estimate for children aged 1 to 4 years as this group was the smallest. We considered the following assumptions: 70% of children would receive two doses of vaccine, alpha error of 5%, absolute precision of 7% for Boffa and 10% for Forécariah, design effect (deff) of 3.0 for Boffa and 1.5 for Forécariah (coverage was expected to be more homogenous in the islands). Taking into account the results of the 2005 Demographic and Health Survey [15], we expected 0.7 children 1–4 year old per household (average of 6.1 individuals per household and 12% of the population between 1 and 4 years). Assuming 10% of missing data, we planned to visit 780 households (60 clusters of 13 households) in Boffa and 180 households (30 clusters of 6 households) in Forécariah. A household was defined as a group of people sleeping under the same roof and sharing meals every day for at least the previous two weeks.

### Training and Data Collection

All surveyors and supervisors were recruited locally and received a theoretical and practical training. Training consisted of survey and interview methodology and a pilot implementation of the questionnaire.

Teams conducted face-to-face interviews after consent. Survey teams asked for the help of neighbours to trace absentees and re-visit empty (but not abandoned) households later in the day. If during the second visit the occupants could not be found or if they refused to participate, that household was skipped.

A standardized pre-piloted questionnaire was used to collect the following information: demographic data (age, sex, and household size), vaccination status (card-confirmed and orally reported), reasons for non-vaccination (open question), and acceptability data (adverse events, taste and beliefs about the vaccine). Questions concerning acceptability were only collected in Boffa (first site of vaccination) in participants older than 15 years. Interviews were conducted in the local language.

### Surveillance of Adverse Events following Immunization

Surveillance of adverse events following immunization (AEFI) was implemented at vaccination sites, health centres and health posts in the target areas. An AEFI was defined as a medical occurrence detected by the vaccination site supervisor or a physician with an onset up to 14 days after receipt of a dose of vaccine. During the awareness campaign and at the time of vaccination, participants were told to report to a vaccination site or a health centre if they felt ill after receiving the vaccine. The following data were collected using a standardized form: age, sex, pregnancy, history of allergies, vaccination date, consultation date, date of onset of the symptoms, type of symptoms, and clinical outcome (recovery, transfer or death).

### Data Entry and Analysis

Our main outcome was the OCV coverage (single dose and full course) in each of the target locations. Vaccine coverage was calculated dividing the number of individuals reporting being vaccinated by the survey population and expressed as a percentage. Vaccination coverage estimates include both card-confirmed and oral reporting. Secondary outcomes included vaccine coverage by age group, sex and reasons for non-vaccination. Crude vaccination coverage estimates and 95% confidence intervals (95% CI) were obtained considering the survey design. The design effect was calculated to estimate the loss of precision due to the cluster based sampling strategy. Sampling weights were calculated to account for differences in the cluster size.

Data entry was performed using EpiData 3.1 (EpiData Association, Denmark) and data analysis was performed using Stata 12.0 (College Station, USA).

### Ethical Considerations

The Ethical Review Board of Guinea and the MSF Ethical Review Board approved the study protocol. Oral informed consent was obtained from participants in all instances. All children had consent given from a parent/guardian and all adult participants provided their own consent. Oral informed consent was requested since the study did not present any risk of harm to subjects and did not involve procedures for which written consent is normally required outside the research context. The procedure was approved by the ethical review boards. The request of consent was registered in a log-book. Privacy and confidentiality of the data collected from participants was ensured both during and after the conduct of the surveys. All treatment was provided free of charge and participation was voluntary.

## Results

**Figure 3:**
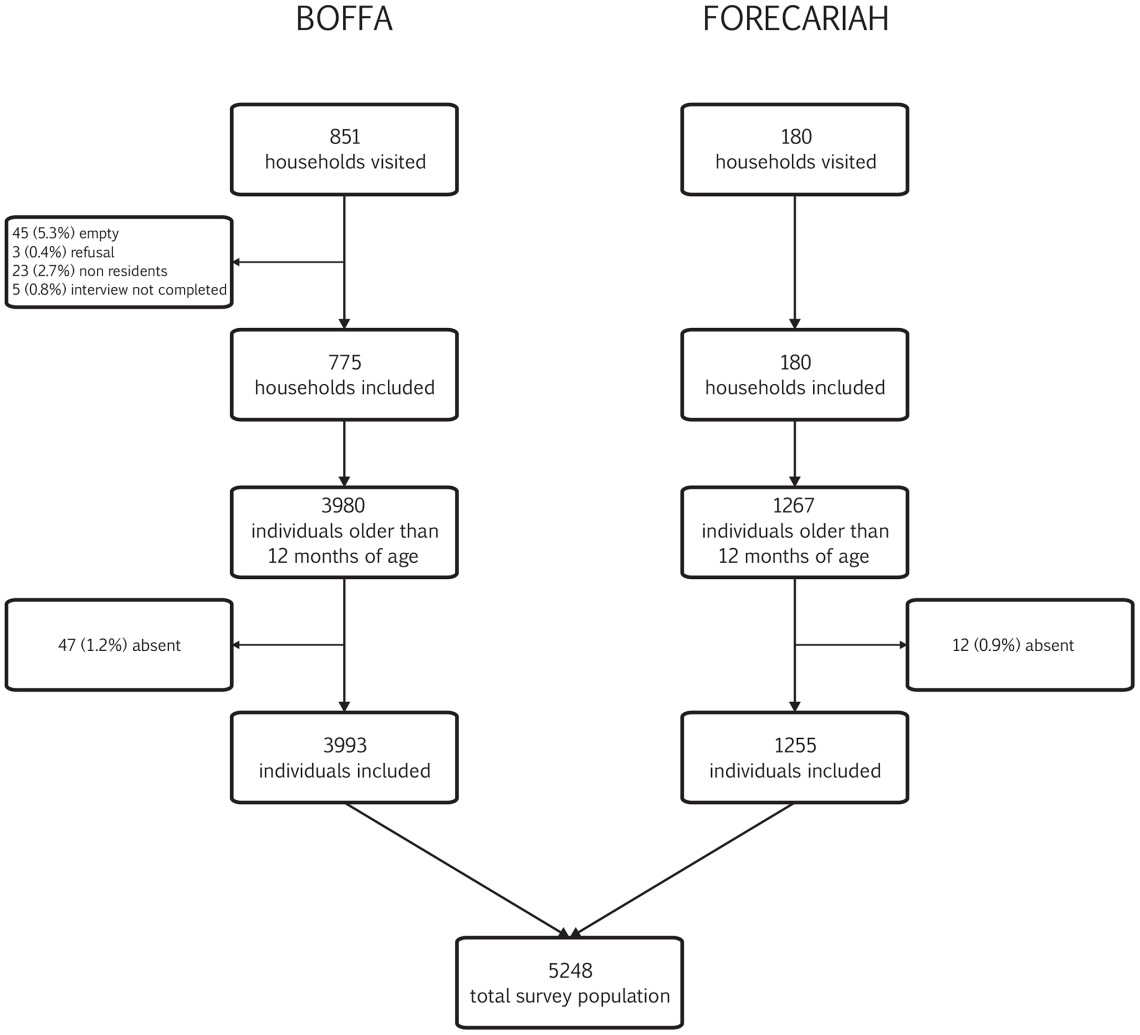
Study flow chart: Number of households visited, number of households included, number of individuals in the targeted age group (older than 12 months of age) residing in the households included in the survey and final number of individuals included in the study.

The surveys were carried out May 20 to 25, 2012 in Boffa and June 16 to 20, 2012 in Forécariah (Figure 2). In total, 851 households were visited in Boffa. Of these, 775 (91.1%) were included in the survey, 45 households (5.3%) remained empty after two visits, 3 households (0.4%) refused to participate and 23 (2.7%) were not residents of Boffa. All 180 visited households were included in Forécariah. Overall, 3,993 individuals were included in Boffa and 1,255 in Forécariah (Figure 3). The median age of participants was 15 years (inter-quartile-range (IQR): 5–30). There were fewer males than females in the survey sample (47.6% and 44.1% males in Boffa and Forécariah respectively).

### Oral Cholera Vaccine Coverage

Vaccination card retention was higher for children (81.7%) than adults (74.8%), and higher for females (82.4%) than males (73.2%).

**Figure 4:**
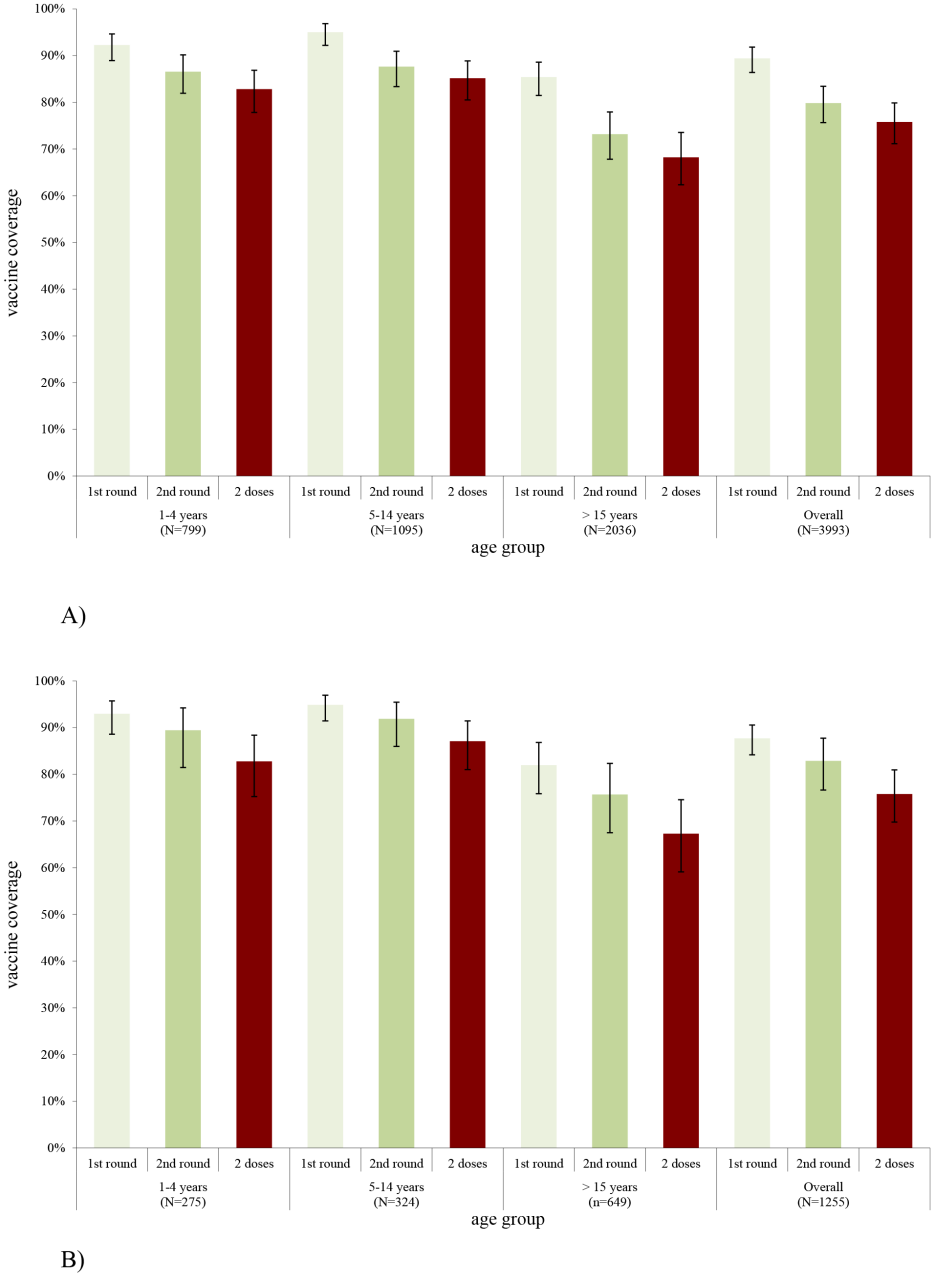
Vaccine coverage by age group of the cholera mass vaccination campaign in Boffa (panel A) and Forécariah (panel B) prefectures, first round, second round and two doses (fully vaccinated), April–June 2012.

Overall, 89.4% [95%CI: 86.4–91.8%] and 87.7% [95%CI: 84.2–90.6%] were vaccinated during the first round and 79.8% [95%CI: 75.6–83.4%] and 82.9% [95%CI: 76.6–87.7%] during the second round in Boffa and Forécariah respectively. The two dose (fully vaccinated) vaccine coverage (including card and oral reporting) was 75.8% [95%CI: 71.2–79.9%, deff = 10.1] in Boffa and 75.9% [95%CI: 69.8–80.9%, deff = 5.0] in Forécariah. Considering incomplete vaccination, 93.3% [95%CI: 91.1–95.0%, deff = 5.9] received at least one dose in Boffa and 94.9% [95%CI: 91.8–96.9%, deff = 3.7] in Forécariah. The dropout rate between the first and second dose was 15.2% [95%CI: 12.2–18.7%] and 13.6% [95%CI: 9.7–18.7%] in each site respectively. Vaccine coverage was lowest among adults in both prefectures (Figure 4).

Vaccine coverage with two doses was similar among females and males in Boffa (76.6% [95%CI: 71.9–80.7%] vs. 75.0% [95%CI: 69.8–79.4%]), but higher among females in Forécariah (79.4% [95%CI: 74.4–83.6%] vs. 71.4% [95%CI: 63.3–78.3%]). Vaccine coverage among women in childbearing age (15–49 years old) was statistically higher than among men of same age in Forécariah (72.6% [95%CI: 65.4–78.8%] vs. 53.4% [95%CI: 41.6–64.8%], p<0.001), but not in Boffa (70.1% [95%CI: 63.8–75.7%] vs. 64.3% [95%CI: 56.1–71.7%], p = 0.1). No major differences were observed in vaccination coverage by sub-prefecture (Table 1).

**Table 1:** Vaccine coverage by sub-prefecture of the cholera mass vaccination campaign in Boffa and Forécariah prefectures, first round, second round and two doses (fully vaccinated), April–June 2012.

	First round		Second round		Full coverage (two doses)	
	n/N (%)*	[95% CI]	n/N (%)*	[95% CI]	n/N (%)*	[95% CI]
Boffa prefecture						
Boffa (n = 850)	773/847 (91)	[82–96]	692/847 (82)	[74–89]	655/847 (78)	[68–86]
Douprou (n = 535)	477/534 (88)	[81–93]	428/534 (79)	[70–86]	411/534 (76)	[67–83]
Koba (n = 957)	835/949 (88)	[83–92]	672/947 (71)	[62–80]	645/946 (69)	[59–77]
Mankountan (n = 577)	535/577 (93)	[88–96]	506/577 (89)	[82–93]	484/577 (84)	[76–90]
Tamita (n = 203)	190/203 (93)	[85–97]	165/202 (80)	[71–87]	160/202 (78)	[66–86]
Tougnifili (n = 811)	725/811 (88)	[77–94]	676/811 (83)	[73–89]	636/811 (77)	[64–86]
Forécariah prefecture						
Kaback (n = 754)	657/744 (87)	[84–90]	605/744 (80)	[72–86]	565/744 (74)	[67–81]
Kakossa (n = 501)	447/501 (88)	[80–93]	451/501 (88)	[76–93]	88/501 (78)	[68–86]

* The vaccine coverage estimates were weighted considering the study design and the confidences intervals were adjusted by the design effect.

Regarding the awareness campaign, 95.7% of survey participants [95%CI: 94.2–96.8%] reported being aware of the campaign. Among individuals not vaccinated, the main reason was “absence during the campaign” for both the first and second rounds. The second most reported reason was “not having time to go for the vaccination” and the third, “sick during the campaign” (Table 2).AEFI was reported as the reason for non-vaccination by 0.9% of non-vaccinated individuals during the second round. A small percentage of participants considered that the vaccine made them feel sick (3.9% [95%CI 2.4–4.7%]). A large proportion of participants reported that the taste of the vaccine was bad (77.6% [95%CI 69.5–84.1%]). Among those vaccinated 1.4% [95%CI: 0.8–2.2%] reported spitting out or vomiting the vaccine. However, 98.9% [95%CI 97.8–99.5%] reported that they would be vaccinated again in a future cholera campaign.

**Table 2:** Reason for non-vaccination among individuals not vaccinated, Boffa and Forécariah prefectures, April–June 2012.

	1st round		2nd round	
	N = 521		N = 952	
Reason	n	%	n	%
**Impossibility to go to the vaccination site**				
Absent during the campaign	411	78.89	672	70.59
The person did not have the time to be vaccinated	30	5.76	81	8.51
Sick during the campaign	24	4.61	42	4.41
The person was hospitalized at the time of vaccination	3	0.58	3	0.32
**Lack of information**				
Not informed about the campaign	17	3.26	28	2.94
The person did not know the date of the campaign	3	0.58	26	2.73
The person did not know the place of vaccination	1	0.19	2	0.21
The caregiver thought that the child was too young	8	1.54	8	0.84
The person thought that he/she was too old	4	0.77	4	0.42
The person thought that one dose was enough	0	0.00	2	0.21
**Logistic constraints**				
Vaccination site considered too far	3	0.58	5	0.53
No vaccines available at the vaccination site	0	0.00	8	0.84
Waiting time too long	0	0.00	8	0.84
**Refusals**				
Cultural beliefs	1	0.19	1	0.11
Bad experience with previous vaccinations	1	0.19	8	0.84
Adverse events during the first round	0	0.00	8	0.85
The vaccine was considered dangerous	0	0.00	1	0.11
**Other**	11	2.11	34	3.57
**No explanation**	4	0.77	11	1.16

### Surveillance of Adverse Events following Immunization

Overall, 48 patients (15 per 100,000 vaccinated) spontaneously reported symptoms that were linked with the vaccine by the health personnel and considered as AEFI with 35 (20 per 100,000 vaccinated) after the first round and 13 (9 per 100,000 vaccinated) after the second round. In total, 29 were women (60%) and the median age was 2**7** years (IQR: 16–36 years); 8 (17%) were children 1 to 4 years. Seven patients reported having a history of allergies (15%). The cause of the allergy was specified for two patients (quinine and chloroquine). The average delay between vaccination and symptom onset was 24 hours with a median delay of 7 hours (IQR: 1–24 hours). One quarter reported the symptoms in the following hour after vaccination. Symptoms reported (n = 139) were mainly gastro-intestinal: 28 (20%) diarrhea, 22 (16%) vomiting, 14 (10%) stomachache and 12 (9%) nausea. In addition, 15 patients (11%) reported fever and general weakness. No patient was transferred to a hospital and no deaths were reported.

## Discussion

The high coverage and good acceptability of the campaigns, conducted in a rural mobile population in Guinea, is encouraging. The percentage of people reporting AEFIs was low and almost all participants reported that they would be vaccinated in a future campaign. However, more evidence is needed about the feasibility of reactive campaigns from densely populated urban scenarios where cholera burden is high and cholera outbreaks evolve faster [16]–[20]. Also the acceptability of target campaigns in such a context should be assessed from a political, public health and community point of view. Determining the short-term protection given by the first dose is a clear priority as an effective one-dose regimen would facilitate the ease and timeliness of reactive campaigns in all contexts.

There are several key limitations of note. Despite the short time span between the vaccination campaign and the data collection for the surveys, we were not able to card-confirm vaccination status for 25% of participants and as a result some information bias may be present. Considering those individuals as not-vaccinated (worst-case scenario), two-dose coverage would decrease to 61% in Boffa and 64% in Forécariah. Second, the precision of estimates was better than expected because the number of participants recruited was higher (linked with the household size composition) than originally planned. However, population estimates in the surveyed areas are likely to be inaccurate. In most areas, no major differences were observed between administrative and survey coverage, but in Kaback an important deviation was observed. Inaccuracies in the population data could have caused some imbalances in the allocations of clusters; as described, we tried to avoid this problem using spatial sampling in Kaback.

An additional limitation concerns the use of a quantitative approach to explore campaign acceptability. Although reasons for non-vaccination were specifically collected using an open question, we cannot exclude the possibility that the population may not have understood certain awareness and education messages. A qualitative assessment would aid in understanding better reasons for non-vaccination, elucidate possible solutions and provide a better understanding of the perception of the vaccination campaigns by the population.

There are few examples where OCVs have been used as public health tools. Dukoral was used pre-emptively in refugee camps in Uganda and Darfur [21], [22] and in endemic areas (Zanzibar and Mozambique) [23], [24]. Shanchol has been recently used in Haiti in a pilot campaign [25]. To our knowledge there are only two published examples of reactive campaigns using OCV, and both were conducted in Asia [26], [27] using vaccines not prequalified by the WHO. The coverage and acceptability of these campaigns varied depending on the setting and the approach (pre-emptive vs. reactive). High coverage was obtained in Uganda, Darfur and Micronesia [21], [22], [26] and lower coverage was obtained in Mozambique, Zanzibar and Vietnam [23], [24], [27]. In Guinea we obtained 76% coverage for two doses and 93% of the population received at least one dose, which represents, to our knowledge, one of the highest coverage reached [21]–[24], [26], [27]. The high coverage obtained is a promising outcome considering that this was one of the largest campaigns conducted in terms of number of doses administered, the specificities of the population (rural and mobile), and the short time available for preparation of the campaign, which has been one of the major arguments against outbreak response with OCV. There are several factors that likely influenced the population to participate in the campaign: first, the campaign was conducted in response to an outbreak and the possibility of even partial protection against a frightening disease was motivating. Second, the population may have been reassured by the involvement of the MoH, public health authorities and MSF; as an example, the vaccination campaign was inaugurated in Boffa with the presence of the Minister of Health. This involvement was also crucial to mobilize human resources and to organize the campaign considering the local specificities. Finally, both the awareness campaign and the vaccination strategy itself (decentralized with sites organized in each village or settlement) involved the communities. This aimed to ensure awareness and provide vaccination opportunities to remote places and difficult to reach population which likely contributed to this high coverage. Vaccination activities started early in the morning and finished late in the afternoon to maximize the opportunities for workers in the main fishing ports. Despite these efforts, the lowest coverage was obtained in adult males.

Significant differences where observed by sex in Forécariah, especially in individuals between 15–49 years old. The vaccination campaign in Forécariah coincided with an intense period in agriculture activities, which was a barrier for the participation in the campaign, especially for the male adults. In addition, the Red Cross Society of Guinea distributed soap and a bottle of chlorine solution to women of childbearing age in Forécariah during the second round of vaccination, which likely increased the coverage in this group. Distribution of soap and chlorine was one of the control measures implemented by the MoH in response to the outbreak in the affected places, but this activity was successfully integrated in Forécariah within the vaccination sites. This suggests that synergies among different preventive approaches is an element to consider in future campaigns both to provide a more comprehensive message on cholera prevention and to improve the vaccine coverage itself.

The number of AEFI reported through the surveillance system was low, without severe AEFI reported. Only a small proportion of non-vaccinated individuals during the second round of vaccination reported AEFI as a cause of non-vaccination. This result is coherent with previous publications on vaccine safety where mild symptoms (mostly not requiring medical attention) have been reported [28], [29]. The proportion of vaccinated individuals reporting AEFIs was lower in our study than in the cluster randomized clinical trial conducted in Kolkata (15 vs. 76 per 100,000) [28]. This difference is probably explained by: first, our surveillance system was passive compared with the active case finding implemented in Kolkata; and second, access to health care was likely more difficult in the vaccinated area in Guinea (remote rural area) than in the urban context of Kolkata.

With respect to the proportion of vaccinees vomiting or spitting out the vaccine after intake, we found a higher percentage than previously documented with Dukoral (no data available for Shanchol) [23]. For administration of Dukoral, the vaccine has to be diluted in water containing a buffer solution. Although administration with water is not necessary for Shanchol, we offered water after vaccine intake. Most vaccinated individuals did not like the taste of the vaccine and offering water may have contributed to fewer incomplete vaccine courses. Additional information should be collected in future campaigns using Shanchol, considering that providing water considerably increased the logistic complexity of the campaign.

In order to facilitate the use of OCV as an additional tool, WHO and partners are in the process of creating a vaccine stockpile dedicated to outbreak response [30]. Here, we showed that high coverage can be reached within a few weeks, even in rural areas, and that the campaigns were well accepted by the population. Good documentation of these interventions is essential to elucidate the strategies leading to successful outcomes as well as key implementation barriers. Synergies between different axes in cholera control interventions should be pursued and other examples of integrated cholera response than the one presented here should serve also to determine the best use of vaccines for cholera prevention and control.

## Supporting Information

Checklist S1 STROBE checklist.
(PDF)
